# Advanced framework for epilepsy detection through image-based EEG signal analysis

**DOI:** 10.3389/fnhum.2024.1336157

**Published:** 2024-01-22

**Authors:** Palani Thanaraj Krishnan, Sudheer Kumar Erramchetty, Bhanu Chander Balusa

**Affiliations:** School of Computer Science and Engineering, Vellore Institute of Technology, Chennai, India

**Keywords:** epilepsy, EEG signal processing, image-based feature extraction, machine learning classifiers, Gramian angular summation field, scale invariant feature transform, oriented FAST and rotated BRIEF

## Abstract

**Background:**

Recurrent and unpredictable seizures characterize epilepsy, a neurological disorder affecting millions worldwide. Epilepsy diagnosis is crucial for timely treatment and better outcomes. Electroencephalography (EEG) time-series data analysis is essential for epilepsy diagnosis and surveillance. Complex signal processing methods used in traditional EEG analysis are computationally demanding and difficult to generalize across patients. Researchers are using machine learning to improve epilepsy detection, particularly visual feature extraction from EEG time-series data.

**Objective:**

This study examines the application of a Gramian Angular Summation Field (GASF) approach for the analysis of EEG signals. Additionally, it explores the utilization of image features, specifically the Scale-Invariant Feature Transform (SIFT) and Oriented FAST and Rotated BRIEF (ORB) techniques, for the purpose of epilepsy detection in EEG data.

**Methods:**

The proposed methodology encompasses the transformation of EEG signals into images based on GASF, followed by the extraction of features utilizing SIFT and ORB techniques, and ultimately, the selection of relevant features. A state-of-the-art machine learning classifier is employed to classify GASF images into two categories: normal EEG patterns and focal EEG patterns. Bern-Barcelona EEG recordings were used to test the proposed method.

**Results:**

This method classifies EEG signals with 96% accuracy using SIFT features and 94% using ORB features. The Random Forest (RF) classifier surpasses state-of-the-art approaches in precision, recall, F1-score, specificity, and Area Under Curve (AUC). The Receiver Operating Characteristic (ROC) curve shows that Random Forest outperforms Support Vector Machine (SVM) and k-Nearest Neighbors (k-NN) classifiers.

**Significance:**

The suggested method has many advantages over time-series EEG data analysis and machine learning classifiers used in epilepsy detection studies. A novel image-based preprocessing pipeline using GASF for robust image synthesis and SIFT and ORB for feature extraction is presented here. The study found that the suggested method can accurately discriminate between normal and focal EEG signals, improving patient outcomes through early and accurate epilepsy diagnosis.

## Introduction

Periodic seizures are a hallmark of the neurological condition known as epilepsy. Abnormal electrical activity in the brain is what causes these seizures. A variety of illnesses or brain injuries can increase the risk of epilepsy. Epilepsy affects an estimated global population of around 50 million individuals (Epilepsy, n.d.; Fiest et al., 2017; Chen et al., 2023). The diagnosis of epilepsy typically involves the assessment of a patient’s medical history, physical examination, as well as the utilization of diagnostic techniques such as electroencephalography (EEG), magnetoencephalography (MEG) and imaging procedures like magnetic resonance imaging (MRI) or computed tomography (CT) scans (Adams et al., 1992). Electrodes are precisely positioned on the scalp in a non-invasive manner in EEG to evaluate and analyze brain activity. In contrast to alternative techniques such as MEG, CT, and MRI, EEG possesses notable merits, including its superior temporal resolution, cost-effectiveness, and widespread availability, rendering it an invaluable instrument (Drenthen et al., 2021). Its remarkable temporal accuracy allows millisecond-scale fluctuations in brain activity to be recorded. For the purpose of identifying dynamic processes and potentials associated with events that are evolving quickly, this degree of temporal resolution is essential. In addition, the EEG is a vital diagnostic tool for epilepsy since it permits real-time tracking of the start, progression, and end of seizures.

Electroencephalography signal processing provides the finest understanding of the dynamic nature of brain illnesses, especially epilepsy (Clarke et al., 2021). However, manual EEG signal analysis and interpretation can be laborious and subjective, primarily depending on the knowledge and skills of neurophysiologists. Because of this, there is an increasing demand for computerized techniques for detecting epilepsy that may automate the analysis process and yield unbiased, precise results (Sharma et al., 2019). In order to aid in the analysis and interpretation of EEG signals, computerized techniques for the detection of epilepsy have been created. These techniques identify EEG signals as either normal or symptomatic of seizure activity by extracting key features using powerful signal processing algorithms and machine learning techniques. Machine learning algorithms are used by computerized epilepsy detection systems to automatically analyze EEG signals and categorize them into seizure and non-seizure categories (Acharya et al., 2013).

Automated epilepsy identification can be greatly aided by common signal processing techniques based on EEG signal classification and machine learning detection. The goal of these techniques is to reliably identify seizure activity from EEG signals by experimenting with different feature extraction and signal classification algorithms. In order to identify epileptic seizures from EEG signals, time-domain techniques have been developed (Fasil and Rajesh, 2019; Chakrabarti et al., 2020). These methods use the temporal properties of EEG recordings to separate normal brain activity from seizure activity (Andrzejak et al., 2001; Tessy et al., 2017). There are also several methods that rely on measures of complexity, like the entropy of an EEG signal (McSharry et al., 2003). They entail calculating the EEG signal’s complexity or unpredictability in order to identify anomalies connected to seizure activity (Acharya et al., 2012; Paul, 2018). Another method for extracting features from EEG recordings to detect epilepsy is the use of local binary patterns (Kumar et al., 2015). With these techniques, local variations in EEG signals are recorded in order to spot patterns suggestive of seizure activity (Kumar and Kanhangad, 2018).

In order to detect epileptic seizures, frequency-domain-based characteristics derived from the Fourier transformation of EEG signals are also essential (Srinivasan et al., 2005). The Fourier transform is a frequently used method for signal processing and feature extraction because it can change a signal from the time domain to the frequency domain. Frequency-domain characteristics derived from the Fourier transform have shown promise in the field of epilepsy detection for detecting seizure activity in EEG recordings (Khamis et al., 2013; Samiee et al., 2015). These characteristics offer crucial details regarding the frequency composition of the EEG signal, which can aid in differentiating between seizures and regular brain activity. These characteristics reveal details about the frequency content of the EEG data, including whether aberrant high-frequency oscillations or seizure-specific rhythmic patterns are present. Algorithms can differentiate between seizure activity and regular brain activity by examining these frequency-domain characteristics, which helps with the precise identification and diagnosis of epilepsy (Tsipouras, 2019).

The Fourier transform is a useful tool for long-term study and real-time monitoring of epileptic seizures because it makes enormous amounts of EEG data computationally and analytically economical. Due to the non-stationary and non-linear nature of EEG signals, complex signal processing techniques are needed, and a key tool for managing these complexities is the Fourier transform and other advanced multiresolution signal processing methods. Moreover, the combination of non-linear parameters calculated from EEG signals with time-frequency domain-based techniques like wavelet transform (Nishad et al., 2020) and empirical mode decomposition (Oweis and Abdulhay, 2011; Cura et al., 2020) adds new dimensions to the categorization of EEG data related to epileptic seizures (Liu et al., 2012; Bajaj and Pachori, 2013). These methods increase the precision of epilepsy identification by accounting for the temporal, frequency, and non-linear properties of EEG signals (Tzallas et al., 2009; Musselman and Djurdjanovic, 2012; Sharma and Pachori, 2015; Li et al., 2018; Wu et al., 2020; Dutta et al., 2023).

Another popular method for detecting epilepsy from an EEG signal is to turn the time series into images for additional examination (Fu et al., 2014; Li et al., 2020; Ozdemir et al., 2021). Among these is the transformation of EEG signals into scalograms or spectrograms for feature extraction and visualization (Acharya et al., 2013; Gómez et al., 2020; Liu et al., 2023). An interesting alternative for epilepsy diagnosis is the use of computer vision techniques in EEG signal analysis, which enables the use of machine learning and pattern recognition algorithms to find unique patterns or anomalies in the EEG data (Bajaj et al., 2017; Zhou et al., 2018). Computer vision-based algorithms for finding epilepsy in EEG signals depend on how well the temporal information of the EEG is turned into spatial information in the form of images (Yogarajan et al., 2023; Zeng et al., 2023). This is because EEG signals change over time. In order to enable the application of modern advanced image processing and machine learning techniques for precise and effective epilepsy detection, we have therefore taken on the task of improving picture-based EEG analysis. We have used a method in this work to encode the EEG signal into images using the GASF (Wang and Oates, 2015; Alsalemi et al., 2023), which is known to preserve the temporal relationships of time series signals (Wang and Oates, 2015; Alsalemi et al., 2023). The application of GASF-based image feature extraction helps to enhance the identification of epilepsy in EEG time-series data is a key component of this work. This evolution may enable earlier diagnosis and treatment, which could improve clinical practice and the quality of life for those with epilepsy. As a result, this work presents a unique method for detecting epilepsy using EEG time-series data and accomplishes its goals by utilizing GASF-based image feature extraction.

Understanding the growing importance and potential of computerized techniques in the detection of epileptic seizures, there is a constant need for innovation and development in this field. The integration of advanced signal processing techniques, such as the GASF, provides a promising approach to further enhance the accuracy and efficacy of epilepsy detection from EEG time-series data. By leveraging GASF-based image feature extraction, this proposed technique aims to preserve the temporal relationships of EEG signals, thereby improving the identification of epilepsy.

### Objectives

In light of these advancements and the evolving landscape of epilepsy detection methods, the objectives of this work encompass the exploration of the GASF-based approach for EEG signal analysis, the assessment of its efficacy in identifying epilepsy, and the potential impact of this method on clinical practice and patient outcomes. This paper endeavors to demonstrate the effectiveness of GASF-based image feature extraction in automating the detection of epilepsy and contributing to early diagnosis and treatment. The specific objectives of this work include:

1. Investigating the GASF as a state-of-the-art technique for obtaining visual features for the detection of epilepsy in EEG time-series data.
2. Analyzing the effectiveness of the two well-known image feature extractors, SIFT and ORB in detecting epilepsy in EEG data when applied to GASF images.
3. Evaluating the accuracy and reliability of epileptic seizure detection from EEG data using GASF-based image feature extraction vs. more traditional methods.

## Related work

Epilepsy, a prevalent neurological disorder with a global impact, is distinguished by the occurrence of recurrent and unpredictable seizures. The precise identification of epilepsy is of utmost importance in order to promptly administer treatment and enhance patient outcomes. The analysis of EEG time-series data is a crucial component in the diagnosis and monitoring of epilepsy. The analysis of traditional EEG typically depends on intricate signal processing techniques, characterized by high computational demands and limited generalizability across diverse patient populations. Given the aforementioned difficulties, scientists are increasingly turning to machine learning methods, particularly those concerning the extraction of diverse features from EEG time-series data, with the intention of enhancing the epilepsy diagnosis process. This literature review examines the field of feature extraction for the purpose of detecting epilepsy. It utilizes machine learning classifiers in conjunction with the publicly accessible EEG database. An extensive range of pertinent studies, methodologies, and their results are thoroughly examined, providing insights into the advancements achieved in this domain and pinpointing possible avenues for enhancement.

Acharya et al. (2019) conducted a comprehensive exploration of characterization of EEG signals with their study delving into methods for the analysis of focal EEG signals. They employed advanced signal processing and machine learning techniques to distinguish focal EEG signals from non-focal ones. This study is a significant Contribution To The Field, as it provides insights into the challenges and potential applications of focal EEG signal analysis (Acharya et al., 2019). Gupta and Pachori presented an innovative approach employing advanced machine intelligence and signal analysis algorithms to classify focal and non-focal EEG signals. Their method demonstrated high accuracy in distinguishing between these types of signals, offering a promising avenue for epilepsy and neurological disorder diagnosis (Gupta and Pachori, 2019).

Hu et al. (2019) used multifractal analysis and several statistical tools, such as the generalized Hurst exponent, the fluctuation index, the mean, and the standard deviation, to find important features in EEG data. Their results are promising, although these methods have limitations, particularly in capturing non-linear EEG signals and their computational burden when dealing with high sampling rates (Hu et al., 2019). Rahman et al. (2019) looked at hybrid features that are made up of improved composite multiscale fuzzy entropy and autoregressive (AR) coefficients. These are derived from variational mode decomposition (VMD), discrete wavelet transform (DWT), and the VMD-DWT domain. This study finds that stacking support vector machine (SVM) and k-nearest neighbors (KNN) classifiers enhances classification accuracy (Rahman et al., 2019). In a related study, Tuncer et al. (2023) proposed a new feature-based EEG signal classification model, including a local histogram-based feature generation function called the cube pattern. Their results highlighted the high performance of the cube pattern and neighborhood component analysis-based model in EEG signal classification (Tuncer et al., 2023).

Zeng et al. (2019) developed an epileptic seizure identification algorithm incorporating empirical mode decomposition (EMD), permutation-entropy-based spectral representation (PSR), and neural networks. Their study involved decomposing EEG signals into Intrinsic Mode Functions (IMFs) using EMD and computing PSR from each IMF to capture relevant features, ultimately leading to the successful classification of focal and non-focal EEG patterns (Zeng et al., 2019).

Sharma et al. (2020) proposed an automated detection approach for focal EEG signals based on the third-order cumulant function. The method effectively identified the difference between focal and non-focal EEG signals by looking at this higher-order statistical property (Sharma et al., 2020). This makes it more likely that an EEG-based neurological disorder diagnosis can be done automatically. Sairamya et al. (2021) leveraged wavelet packet decomposition and quad binary patterns to automatically detect focal EEG signals. The use of quad binary patterns and wavelet packet decomposition, extracts discriminative features and significantly contributes to EEG-based neurological disorder diagnosis (Sairamya et al., 2021).

Xuyang et al. (2021) explored a multi-feature fusion approach for epileptic focus localization through tensor representation. The fusion of features extracted from EEG signals enhanced the accuracy of epileptic focus localization, offering potential advancements in epilepsy diagnosis (Xuyang et al., 2021). Wang et al. (2021) proposed a computer-aided intracranial EEG signal identification method using a multi-branch deep learning fusion model. This model uses a number of different deep learning architectures to correctly identify intracranial EEG signals, which showed promise for accurate EEG signal identification (Wang et al., 2021).

Borowska introduced a novel multiscale permutation Lempel-Ziv complexity measure for biomedical signal analysis. This method improved the study of focal EEG signals by looking at them at different scales and giving useful information about the data’s complexity and patterns (Borowska and Syczewska, 2021). Zhao et al. (2023) presented a classification method for the epileptic seizure onset zone based on partial annotation. According to Zhao et al. (2023), their method used partial annotations to accurately find the area in EEG data where the seizure starts. This leads to accurate classification and helps with cognitive neurodynamics and epilepsy diagnosis.

In their paper, Al-Salman et al. (2022) suggested using a dual-tree complex wavelet transform (DTCWT) along with a classification algorithm to get information about epileptic features from EEG signals. The DTCWT gets useful frequency-domain data from EEG signals, which lets features of epilepsy be grouped in a way that looks promising (Al-Salman et al., 2022). Sharma (2023) introduced an automated hybrid approach for localizing the epileptic surgical area. This method used higher-order statistics, sensitivity analysis, and residual wavelet transforms to precisely locate the surgical area. This makes it easier to find epileptic areas in EEG signals (Sharma, 2023).

In contrast to the existing approaches for epilepsy identification from EEG signals, our proposed method takes a novel and innovative approach. Our approach distinguishes itself by translating EEG signals into GASF images and employing SIFT and ORB techniques for feature extraction. Whereas the traditional methods typically involve intricate signal processing and direct analysis of raw EEG time-series data. However, those approaches often lead to increased computational overhead and limited capture of spatial patterns. Our methodology bridges this gap by converting EEG data into visual GASF images, allowing us to harness spatial properties that are frequently overlooked in conventional analyses. Feature extraction using SIFT and ORB introduces a new dimension to our work, enabling the identification of significant features within GASF images and providing access to the rich spatial and structural information present in EEG data. This retreat from traditional feature extraction techniques has the potential to reveal previously undiscovered discriminative patterns, ultimately improving classification accuracy and robustness.

An important shift in our approach pertains to the use of machine learning (ML) methods to classify GASF images as representing either normal or focal EEG patterns. This is a departure from conventional methodologies that lack adaptability and struggle with generalization, particularly in diverse patient settings. The technical originality of our approach and its potential therapeutic impact make it a significant contribution. The holistic strategy, which leverages spatial patterns and image-based feature extraction, holds the promise of improving epilepsy diagnosis accuracy, facilitating faster interventions, and ultimately enhancing patient outcomes. The adaptability of our approach to diverse EEG patterns and patient demographics is crucial for personalized neurological condition diagnosis. This innovation opens new horizons in EEG-based epilepsy detection, augmenting diagnostic precision, and the field of neurological disorder research.

## Materials and methods

Muscle activity and external disturbance can disrupt EEG signals. Noise from these sources can impair EEG signals and make neurological diagnosis difficult. To address this issue, researchers are developing more robust EEG signal representations that preserve temporal correlations and improve diagnostic accuracy. EEG signals can be converted into GASF pictures. The GASF time series representation method uses cosine and sine modifications to turn a sequential data stream like the EEG signal into a picture. GASF images reduce noise while preserving EEG temporal correlations. By extracting GASF images from EEG signals, we can use image processing and analysis for classification.

These GASF images are processed with robust image feature extraction methods like SIFT and ORB to find significant patterns and discriminative features. After applying these reliable image feature extractors, we can efficiently examine GASF images and classify them into neurological or cognitive states. Thus, EEG signal transformation to GASF pictures, followed by image feature extraction and classification, can increase EEG-based diagnosis accuracy and trustworthiness. The proposed method also improves noise handling and brain activity pattern recognition for specific situations or states.

### Dataset

The Bern-Barcelona EEG database, a valuable resource for epilepsy-related studies, provides a diverse collection of EEG recordings from patients with epilepsy during seizure and normal states. This database includes EEG data from different age groups, seizure types, and various clinical scenarios, making it suitable for training, validating, and testing machine learning models for epilepsy detection. The database was created by Andrzejak et al. (2012) and was published (The Bern-Barcelona Eeg database - Nonlinear Time Series Analysis (UPF), n.d.). The database comprises a total of 7,500 signal sets, which have been classified into two distinct categories, namely normal signals and focal signals. Non-focal signals are derived from the typical regions of the brain, while focal signals are derived from the specific brain areas observed during visual inspection of an ictal event. The database is commonly used to test and validate computer-aided diagnosis (CAD) systems for epilepsy detection. Research on epilepsy may benefit greatly from the Bern-Barcelona EEG Database, especially when examining the properties of focal and non-focal EEG signals. Below is a summary of its salient features:

### Subject count

• Five people with epilepsy have their recordings in the database.

### Diversity

• Age range: at the time of recording, 24–42 years old.
• Men: three, women: two.
• Type of seizure: All patients had long-standing, drug-resistant temporal lobe epilepsy.
• EEG recordings are made for a maximum of 7 h, with several recordings made for each patient.
• A total of 64 electrodes are used in the intracranial EEG (iEEG) recording system.

### Potential biases

• Small sample size: The results limited capacity to be generalized is hampered by the small number of individuals.
• Uniform group: The emphasis on medication-resistant temporal lobe epilepsy restricts the relevance of the findings to other forms of epilepsy or to more general neuroscience studies.
• Limitations related to age and gender: The study’s emphasis is on a particular age group and gender ratio, which may restrict its applicability to larger age groups or other gender ratios.
• Extracranial vs. Intracranial EEG: Although iEEG data is more intrusive and may not accurately reflect scalp EEG recordings utilized in clinical settings, it does give a better spatial resolution.

Particular reasons for selecting the Bern-Barcelona EEG Database over alternatives are:

### Relevance

• Emphasis on iEEG: Compared to scalp EEG, high-resolution intracranial EEG (iEEG) recordings give more accurate spatial information. Studying focal epilepsy, in which seizures start in certain brain areas, is critical.
• Drug-resistant temporal lobe epilepsy is a frequent and difficult kind of epilepsy that is the subject of this database. This makes it possible for researchers to look at certain traits and patterns associated with this kind of epilepsy.
• Long-standing epilepsy: Compared to freshly diagnosed instances, the patients who were chosen had long-standing epilepsy, which enhances the chance of recording a variety of interesting seizure patterns.

### Representativeness

• Comparing intracranial and extracranial EEG reveals that while the former has benefits, the latter is an invasive process unsuitable for therapeutic use. By supplying ground truth iEEG data that can be utilized to evaluate and interpret scalp EEG results, the database serves as a useful link between iEEG and scalp EEG research.
• Each participant has numerous recordings in the database, which captures diversity in seizure patterns and may improve generalizability.
• Because the database is publicly accessible, researchers may collaborate and advance their work more quickly by duplicating and expanding upon earlier discoveries.

**FIGURE 1:**
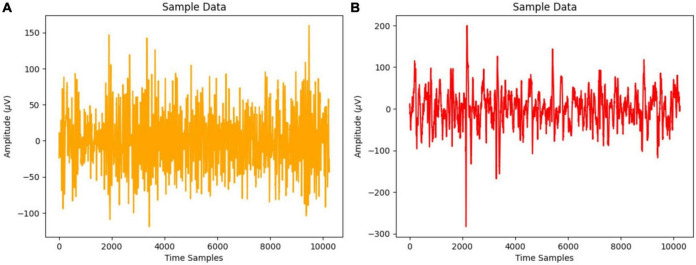
**(A)** Normal waves and **(B)** focal waves on a single channel EEG signal.

Figure 1 displays the EEG signal time series representation for normal and focused participants using a 20-s time frame that yields 10,240 samples at 512 Hz sampling frequency.

**FIGURE 2:**
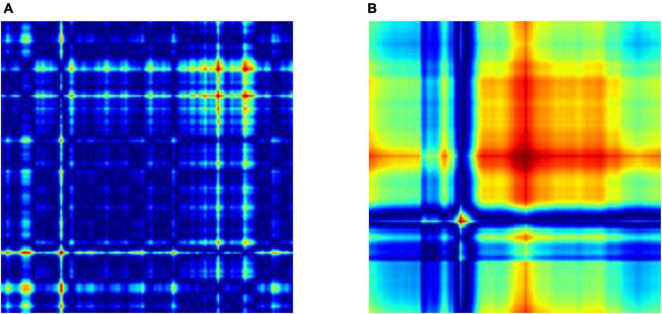
Two types of EEG signals, Normal and Focal, shown using GASF after the data was split: **(A)** normal GASF and **(B)** focal GASF. Since 256 samples are used to make the GASF image, its size is 256 × 256.

Figure 2 displays the GASF of the EEG epochs which are segmented into 256 samples. It leads to a GASF image of dimension 256 × 256 as shown in Figure 2 Moreover, we could also observe that the image shows a difference in the field strength for the normal and focal EEG signals, respectively. For the experimental work in this study, we have selected 50 normal and 50 focal EEG signals provided in the dataset repository. We have transformed each EEG signal into 40 GASF images considering 256 samples. Thus, we have created a total of 4,000 GASF images for both the classes. Out of which we have used 90% (3600 GASF images) for training and validation and the remaining 10% (400 GASF images) for testing the proposed method.

### Methodology

Electroencephalography is essential for diagnosing neurological diseases and brain activity. However, EEG readings include both appropriate cerebral activity and unwanted distortions, making it difficult to distinguish between normal and focal data. This work proposes a complete preprocessing strategy to address these difficulties. The suggested pipeline includes data partitioning, GASF signal transformation into image-like representations, SIFT and ORB image-based feature extraction, and feature selection. To maintain unbiased reporting, the EEG dataset is split into training and testing sets. GASF converts EEG signals into images for SIFT and ORB feature extraction. These image-based methods reveal key patterns for identifying normal from focal EEG data. Later feature selection keeps useful features, improving machine learning classifier performance.

**FIGURE 3:**
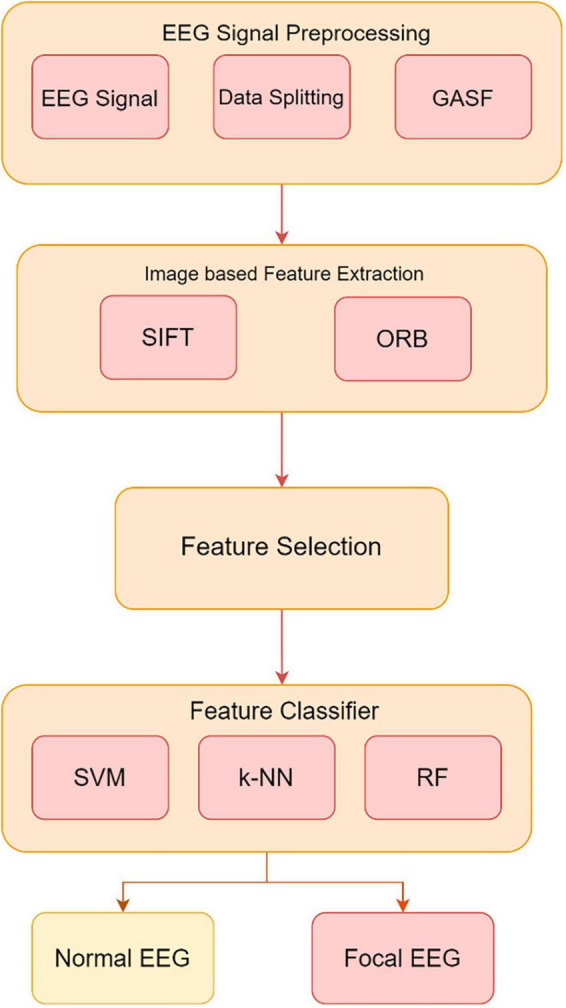
Method proposed for developing an image-based system utilizing the GASF images to extract and classify features from EEG signals.

Experimental validation uses EEG data with Support Vector Machine (SVM), k-Nearest Neighbor (k-NN), and Random Forest (RF) classifiers trained on extracted features. In Figure 3, the GASF-based epilepsy detection system proceeds from data partitioning to GASF image production, SIFT and ORB image-based feature extraction, and feature selection. This preprocessing pipeline improves EEG-based classification accuracy and efficacy, promising neurological disease detection.

### Formation of GASF images from time series signals

To convert time-series signals into temporal images, Wang and Oates proposed GASF where the input time series data is normalized within the range [−1, 1] before being encoded (Wang and Oates, 2015). Then through scaling, the time-series signal is transformed from a Cartesian coordinate to a polar coordinate thus preserving the input signal’s temporal information. Next, the temporal association between the discrete-time points in the polar coordinates are established through the application of the trigonometric cosine function, resulting in a *n*×*n* dimensional Gramian matrix. Here, “*n”* denotes the sample points for the EEG time period. This could be further explained as follows:

Let *T* = {*t*_1_,*t*_2_,…,*t*_*n*_} be a signal with “n” samples, and T be rescaled to have an interval of [−1, 1]. This can be shown in the following way Eq. 1:


(1) $$T_{0}^{∼i}=\frac{t_{i}-m\,i\,n\,\left(T\right)}{m\,a\,x\,\left(T\right)-m\,i\,n\,\left(T\right)}$$

After that, the equation below is used to find the angle φ as shown in Eq. 2.


(2) $$\varphi =\arccos\tilde{(}T_{o}^{i})$$

By adding up the angles of two points next to each other, “i” and “j,” we can find their temporal correlations. This gives us the Gram matrix called GASF. This can be written as in Eq. 3:


(3) $$G\,A\,S\,F=\left[c\,o\,s\,\left(\varphi_{i}+\varphi_{j}\right)\right]$$

Using this method, we can turn a specific time-series sample into an image. This procedure allows for the extraction of important features from the EEG data, which can then be used for further analysis and classification. Additionally, GASF provides a compact representation of the EEG signals, reducing the computational complexity and memory requirements of the analysis process. The Algorithm 1 summarizes the procedure of GASF.

**Algorithm 1:** Computation of GASF.

*Input:N epochs EEG Signal of length, I =256 samples* *Output:GASF Matrix* *Initialize square matrixGAFS[256,256]* *for epoch→1, N do* *Min - Max normalization of the time series data [−1,1]* *Convertion of each time series ponts into Polar Co – ordinate* *for time point →1, l do* *Compute Correlation of the two ponts:cosine of the sum of* *the angles of the two points* *end for* *end for*

Once these steps are completed, we will have a GASF image that represents our time series signal. This image can then be used as input to machine learning algorithms for tasks such as classification.

### Image-based feature extraction with SIFT and ORB

Our preprocessing pipeline leverages image-based feature extraction with SIFT and ORB algorithms. The reason for selecting these image-based features are their ability to capture local and distinctive information from the EEG signals. The rationale behind choosing these image-based features lies in their capability to encapsulate localized and unique information from the EEG signals. The robustness and adaptability of the SIFT and ORB algorithms make them suitable choices for capturing intricate details within the EEG signals, thereby enriching the feature set used for further analysis and classification. These methods identify keypoints in the EEG image, representing distinctive patterns in the signal. By capturing scale-invariant and rotational-invariant features, SIFT and ORB allow for robust feature extraction, enabling accurate classification of normal and focal EEG signals.

### SIFT feature extraction procedure

**FIGURE 4:**
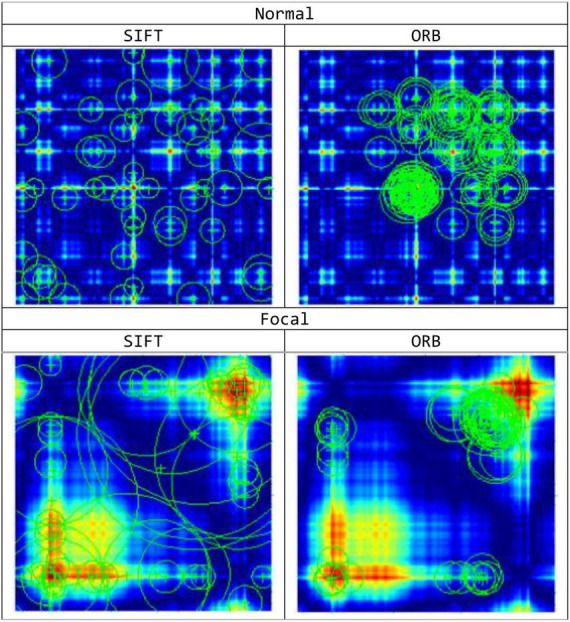
Feature Keypoint Representation based on SIFT and ORB feature extraction methods for the normal and focal GASF images.

The four steps of the SIFT algorithm are described in more detail below (Lowe, 2004):

1. Scale-space peak selection: This step involves constructing a scale space to detect potential locations for finding features. The Laplacian of Gaussian (LoG) is computed for the image using different standard deviation (σ) values. The LoG serves as a blob detector, capable of detecting blobs of different sizes by utilizing variations in the scaling parameter, σ. Nevertheless, due to its high computational cost, the SIFT algorithm employs the Difference of Gaussians (DoG) method as an approximation to the LoG. After the computation of the DoG, a search is conducted on images to identify local extrema across both scale and space. This search aims to identify potential locations of keypoints.
2. Keypoint Localization: After identifying the potential keypoints locations, further refinement is performed to enhance the accuracy of the outcomes. In order to enhance the stability of the remaining keypoints, low-contrast keypoints and edge responses are eliminated during the process. Figure 4 shows the obtained feature points for the normal and focal GASF images based on the SIFT method.
3. Orientation Assignment: The assignment of an orientation is performed for each keypoints based on the local image gradient directions. The operation achieves invariance to rotation.
4. Keypoints Descriptor: The final step involves computing a descriptor for each keypoints that captures the local image information around it. The descriptor is then used for tasks like image matching, object recognition, and image retrieval.

### ORB feature extraction procedure

The ORB feature extraction technique is described in more detail below (Rublee et al., 2011):

1. Convert the image to grayscale: Initially for ORB feature extraction process the input image is converted to grayscale, since like many image processing algorithms.
2. Initialize the ORB detector and detect the keypoints: ORB uses the Features from Accelerated Segment Test (FAST) keypoint detector to detect features in the image. FAST is employed to detect corners and features points in the image. ORB also uses a scale pyramid to produce multiscale features. Figure 4 shows the obtained feature points for the normal and focal GASF images based on ORB method.
3. Compute the descriptors: Once the keypoints have been detected, the next step is to compute descriptors for each keypoint. Descriptors capture information about the local image region around each keypoint and are used for tasks such as matching keypoints between images. For this, ORB uses the Binary Robust Independent Elementary Features (BRIEF) descriptor which takes a smoothed image patch and selects a set of “*nd (x,y)”* location pairs in a unique way. Then, pixel intensity comparisons are done on these location pairs. For example, let the first location pair be “*p”* and “*q*.” If *I(p)* < *I(q)*, then its result is 1, else it is 0. This is applied for all the “nd” location pairs to get an nd-dimensional bitstring. This “*nd”* can be 128, 256 or 512. In this work, we have taken a string length of 128. However, due to the poor performance of BRIEF when rotated, ORB applies a rotation to the BRIEF based on the keypoint orientation.

### Bag of words

**FIGURE 5:**
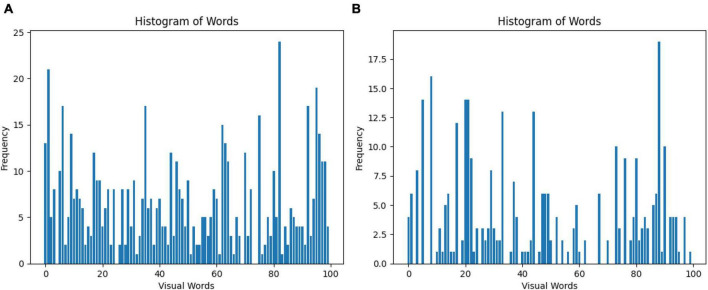
Visual representation of the Bag of Words obtained from the **(A)** SIFT and **(B)** ORB feature extraction step. A total of 100 features are selected for the classification of GASF images.

The Bag of Words (BoW) is a technique for representing images as a set of unordered words, where each word corresponds to a visual feature. In this case, 100 features were selected for the classification of GASF images. Figure 5 shows a line graph with two axes: the x-axis represents the visual words, and the y-axis represents the frequency of each visual word in the image. The higher the frequency of a visual word, the more likely it is that the image contains that visual feature. The BoW representation of an image is used for image classification. By comparing the BoW representation of an image to the BoW representations of a set of training images, it is possible to determine the class of the new image or specifically a normal GASF or an abnormal GASF image.

### Feature selection

**FIGURE 6:**
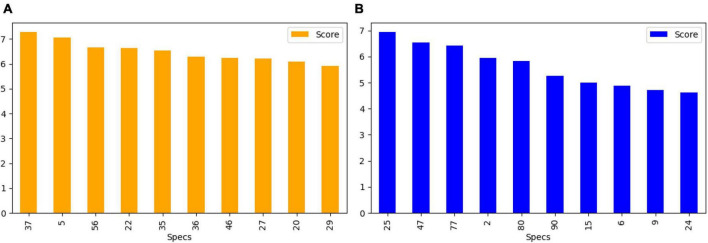
Top 10 features selected **(A)** SIFT **(B)** ORB for the training and validation of state-of-the-art classifiers.

After feature extraction, we perform feature selection to retain the most informative keypoints. This step helps reduce dimensionality, focusing on the most relevant features for EEG signal classification. By retaining the most salient features, we improve the efficiency of our subsequent machine learning classifiers. The chi-square test was chosen for feature selection due to its suitability for determining the association between categorical variables, which is relevant in the context of feature selection for machine learning. This statistical method is particularly well-suited for datasets with categorical features, making it an appropriate choice for the analysis of the extracted features from the EEG data. We calculate the chi-square statistic between each feature and the target variable and select the desired number of features with the best chi-square scores. The idea is to select features that are more strongly associated with the target variable, as indicated by a low *p*-value. Figure 6 shows the selected discriminant features of the SIFT and ORB features extracted from the GASF images. The selected features alone are used for training the machine learning classifiers (Zhou et al., 2013).

### Machine learning classifiers

Support Vector Machine, k-NN, and RF are the machine learning classifiers implemented in this work. This is due to the fact that these classifiers are all widely used and well-established machine learning algorithms that have demonstrated excellent performance in a variety of applications. SVMs are robust classifiers that perform admirably with both linear and non-linear data. It is especially advantageous when confronted with high-dimensional data and possesses the capability to process extensive datasets. Furthermore, SVM functions by identifying the most advantageous hyperplane for class separation, with the objective of maximizing the distance between this hyperplane and the data points that are closest to each class. SVMs utilize support vectors, which are the nearest data points, and employ kernel functions to represent non-linear decision boundaries, making them well-suited for high-dimensional data (Zhou and Li, 2020). In contrast, k-NN is a straightforward and intuitive classifier that excels when the data is represented as points in a space with multiple dimensions. k-NN, which is simple but computationally demanding, operates on the principle of data similarity. The process of binary image categorization involves determining the class of a test example by utilizing the majority class among its nearest neighbors and identifying the “k” training examples that are closest to it in feature space. The selection of the hyperparameter “k” is possible via cross-validation; however, the computational requirements may present difficulties when dealing with large datasets. In contrast, RF is an ensemble learning technique that generates predictions by combining multiple decision trees (Guerrero et al., 2021). RF demonstrates exceptional performance in tasks involving classification or regression. Multiple decision trees and their predictions are utilized. By training each tree on a random subset of the training data and features, overfitting is avoided and generalization is improved. The ensemble methodology effectively handles intricate decision boundaries, data with a high dimension, and outliers.

The application of SVM, KNN, and RF in the context of epilepsy identification from EEG recordings is influenced by a number of factors (Kumar et al., 2014). These classifiers enhance the unique characteristics of the proposed method and offer evident advantages over current state-of-the-art approaches (Kumar et al., 2023).

## Experimental results

**FIGURE 7:**
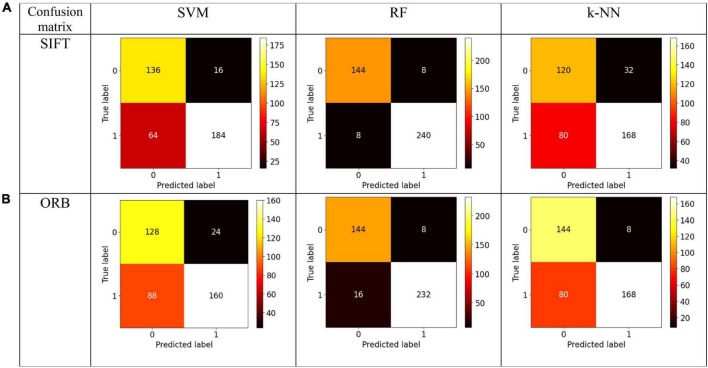
Confusion Matrix for the state-of-the-art classifiers for the **(A)** SIFT and **(B)** ORB features.

We evaluate our preprocessing methodology by analyzing a comprehensive collection of EEG recordings. The performance of the classifier is evaluated for discrimination of normal and GASF-based focal EEG patterns. A number of commonly employed classifier metrics are utilized to evaluate the performance of the three classifiers investigated in this study. Firstly, the confusion matrix for three distinct machine learning algorithms—SVM, RF, and k-NN—is depicted in Figure 7. A table that displays the true and predicted classes for a given set of data is known as the confusion matrix. The confusion matrix comprises numerical values denoting the proportion of accurately classified data points in comparison to the number of misclassified data points. To determine the classifier’s overall accuracy, divide the sum of the data points that were correctly classified by the total number of data points. A valuable instrument for assessing the performance of a machine learning algorithm is the confusion matrix. It can be utilized to ascertain the accuracy of the classifier and identify the classes that are being misclassified. The confusion matrix, as illustrated in Figure 7, indicates that the quantity of accurate predictions, specifically true positives (TP) and true negatives (TN) (represented by the confusion matrix’s diagonal elements), is relatively greater for both features in comparison to the number of false positives (FP) and false negatives (FN) (off-diagonal elements). Nevertheless, additional quantitative metrics are calculated in accordance with the confusion matrix and are presented in Table 1.

**TABLE 1:** Performance of the state-of-the-art classifiers for the SIFT and ORB based features.

Classifier/metrics	Accuracy	Precision	Recall	Specificity	F1-score	AUC	LogLoss
**SIFT**							
SVM	0.80	0.92	0.74	0.89	0.82	0.86	0.14231
RF	0.96	0.97	0.97	0.95	0.97	0.97	0.01274
k-NN	0.72	0.84	0.68	0.79	0.75	0.74	0.24960
**ORB**							
SVM	0.72	0.87	0.65	0.84	0.74	0.83	0.25425
RF	0.94	0.97	0.94	0.95	0.95	0.95	0.02597
k-NN	0.78	0.96	0.68	0.95	0.79	0.81	0.18721

The table provided here (Table 1) shows the performance of different machine learning classifiers on two different feature sets (SIFT and ORB) for image classification. The classifiers are SVM-Radial Basis Function (SVM-RBF), RF, and k-NN. The Table 1 shows the following metrics for each classifier and feature set:

1. Accuracy (ACC): The ratio of correct predictions to the total number of predictions made.
2. Precision (PRE): The ratio of TPs to the number of all positive predictions.
3. Recall (RC): The ratio of TPs to the number of all actual positives.
4. Specificity (SPEC): It measures how well a model correctly identifies TNs out of all actual negatives in the data.
5. F1-Score (F1): A weighted average of PRE and RC.
6. AUC: The area under the ROC curve.
7. LogLoss: Log-loss measures the dissimilarity between the actual target values and the predicted probabilities generated by the classifier.

A higher value for each metric indicates better performance except for Logloss, which measures the discrepancy between the predicted and the actual probabilities. For the SIFT feature set, RF has the highest ACC, PRE, RC, SPEC, F1-score and lowest Logloss value. SVM has the second highest ACC, PRE, SPEC, RC, and F1-score. *k*-NN has the lowest ACC, SPEC, PRE, RC, and F1-score. Likewise, for the ORB feature set, RF has the highest AC, PRE, SPEC, RC, and F1-score. Interestingly, *k*-NN has the second highest ACC, PRE, SPEC, RC, and F1-score while SVM has the lowest ACC, PRE, SPEC, RC, and F1-score. Overall, RF performs the best on both feature sets, followed by SVM and k-NN.

**FIGURE 8:**
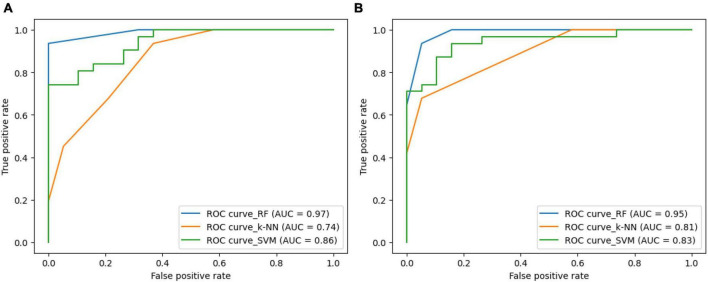
Receiver Operating Characteristic (ROC)-curve for the state-of-the-art classifiers for the **(A)** SIFT and **(B)** ORB features.

Figure 8 illustrates the ROC-curves for the state-of-the-art classifiers for the SIFT and ORB features. A ROC curve is a plot of the true positive rate (TPR) against the false positive rate (FPR). The TPR is the ratio of true positives to the number of all actual positives, and the FPR is the ratio of false positives to the number of all actual negatives. In the Figure 8, the ROC curves for the SIFT and ORB features are shown in (a) and (b), respectively. The SVM classifier is shown in green, the RF classifier is shown in blue, and the *k*-NN classifier is shown in orange.

The ROC curves show that the RF classifier performs the best on both the SIFT and ORB features. The RF classifier has the highest TPR at all FPR values, which means that it is the best at detecting true positives while minimizing false positives. The SVM classifier performs the second best on both the SIFT and ORB features. The SVM classifier has a slightly lower TPR than the RF classifier, but it also has a slightly lower FPR. This means that the SVM classifier is slightly less likely to detect true positives, but it is also slightly less likely to generate false positives. The k-NN classifier performs comparatively less on both the SIFT and ORB features. The k-NN classifier has a much lower TPR than the SVM and RF classifiers, and it also has a much higher FPR. This means that the k-NN classifier is much more likely to generate false positives, and it is also much less likely to detect true positives. Overall, the RF classifier is the best choice for image classification when using the SIFT or ORB features. The SVM classifier is a good second choice, and the k-NN classifier shall be avoided.

The experimental findings indicate that the classification accuracy of our proposed pipeline is considerably enhanced in comparison to baseline methods. The results of our study demonstrate that the implementation of GASF, image-based feature extraction utilizing SIFT, and feature selection significantly improve the performance of EEG signal classification.

### Noise analysis

**FIGURE 9:**
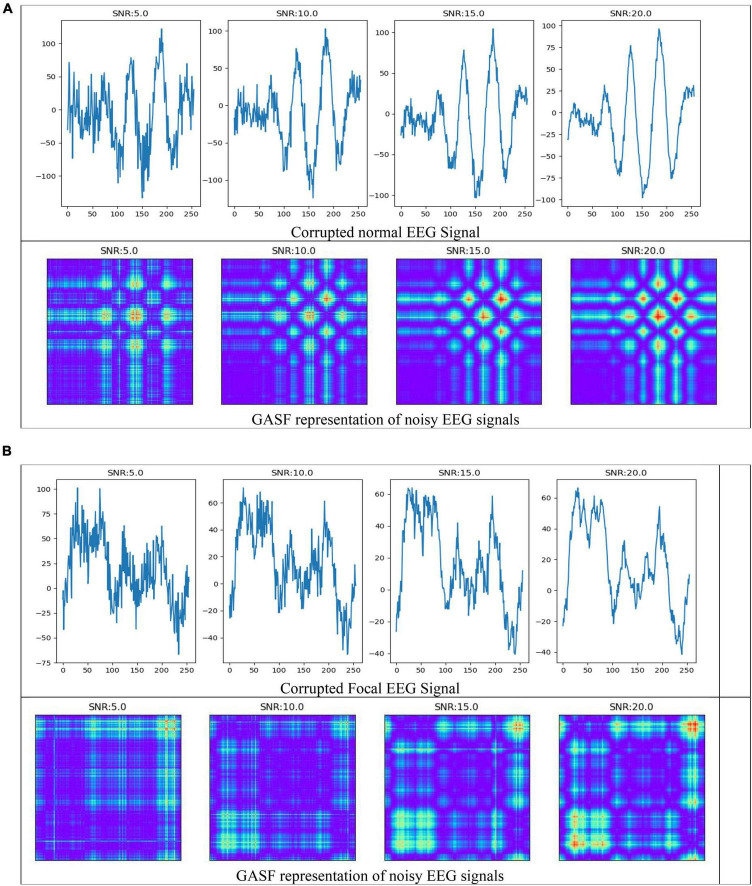
Electroencephalography (EEG) signals corrupted by Gaussian noise and its representation in GASF images **(A)** normal **(B)** Focal signals.

In the field of EEG signal classification, noise is a common problem that can make it difficult to extract meaningful information from the data. In this study, we investigated the effects of Gaussian noise on the accuracy of our proposed method of image-based feature extraction and classification of EEG signals. We first corrupted EEG signals with Gaussian noise at different signal-to-noise ratios (SNRs). We then used GASF to convert the EEG signals to images, SIFT and ORB to extract features from the images, and RF to classify the features. As shown in Figure 9, the GASF images of EEG signals corrupted with noise at high levels (e.g., SNR = 5.0 dB) are significantly distorted compared to the GASF images generated with low noise levels (e.g., SNR = 20.0 dB). We computed the performance metrics of such noise EEG samples (40 normal and 40 focal) using the proposed method and tabulated in Table 2. The table shows the average ACC and F1-score for the SIFT and ORB feature extractors at four different SNRs (5, 10, 15, and 20 dBs). We found that the detection performance decreased as the noise level increased. However, the accuracy remained relatively high even at low SNRs (5 dB). This suggests that EEG signals can still be used to accurately predict epilepsy conditions even in the presence of noise using the proposed system. The results of this study can be used to improve the robustness of EEG-based epilepsy detection algorithms.

**TABLE 2:** Validation of the proposed system for EEG signals corrupted by Gaussian noise at different Signal to Noise Ratio (SNR).

Metrics/SNR	5	10	15	20
**SIFT**				
**Normal**				
Accuracy	93.3	93.8	94.6	95.2
F1-score	93.2	93.4	93.9	94.3
**Focal**				
Accuracy	92.1	93.5	94.1	94.6
F1-score	92.6	92.8	93.7	94.4
**ORB**				
**Normal**				
Accuracy	91.4	91.7	92.1	92.8
F1-score	90.5	91.4	92.6	93.1
**Focal**				
Accuracy	90.1	90.5	91.1	91.9
F1-score	89.3	90.4	91.0	91.6

## Discussion

The field of seizure detection has witnessed significant advancements, as evidenced by the diverse methods presented in this comparative study as shown in Table 3. The evaluated methods employ various feature extraction techniques to enhance the accuracy of seizure detection. In this discussion, we delve into the strengths and limitations of each method, considering their implications for real-world applications and the broader landscape of medical signal processing. It’s important to note that while other schemes used EEG time series directly or transformed to other domains such as Time-Frequency or binary patterns for epilepsy detection, the proposed scheme in our work utilized image-based analysis based on GASF images from the EEG signal. Therefore, a direct comparison between the proposed method and the other schemes is limited due to the difference in the nature of the data used for analysis. However, we have included the results of other schemes for reference and to provide context within the field.

**TABLE 3:** Comparison of the proposed GASF based epilepsy detection and state-of-the art methods on Bern Barcelona EEG dataset.

References	Method	Extracted features	Sensitivity	Specificity	Accuracy
Sharma et al., 2020	Higher order statistics	Statistical features from third order Cumulant	99.33%	98.66%	99%
Sairamya et al., 2021	Quad binary pattern	Entropy-based features	95.74%	95.73%	95.74%
Xuyang et al., 2021	STFT, 1D-CNN	Multi-feature fusion	92.50%	94.38%	93.44%
Wang et al., 2021	Deep fusion	Combination of time, frequency, T-F and entropy features	97.78%	97.42%	97.60%
Zhao et al., 2023	STFT, FCNN	Entropy, frequency domain features	–	–	88.14%
Proposed method	GASF	SIFT/ORB image features	97% (SIFT) 94% (ORB)	95% (SIFT/ORB)	96% (SIFT) 94% (ORB)

The Third Order Cumulant was used by Sharma et al. (2020) to generate statistical features for Higher Order Statistics (HOS). With a sensitivity of 99.33%, specificity of 98.66%, and overall accuracy of 99%, this approach performs quite well. The use of HOS suggests a study of higher-order statistical moments, which can identify complex patterns in the data. This method works very well for identifying minute alterations linked to seizure activity. Nevertheless, real-time applications may face difficulties due to the computational cost of HOS-based approaches, requiring additional optimization. Using entropy-based characteristics, Sairamya et al. (2021) introduced the Quad Binary Pattern technique. The technique yielded an accuracy of 95.74% with balanced sensitivity (95.74%) and specificity (95.73%). An emphasis on capturing local spatial correlations within the signal is suggested by the use of binary patterns. Although the approach works well overall, it may be vulnerable to signal artifacts or noise because of its reliance on entropy properties. More research is necessary to determine how reliable the Quad Binary Pattern approach is in the presence of noise, particularly in situations where the quality of the data may be at risk.

A method combining 1D-Convolutional Neural Network (CNN) and Short-Time Fourier Transform (STFT) with multi-feature fusion was presented by Xuyang et al. (2021). With competitive sensitivity (92.50%), specificity (94.38%), and an overall accuracy of 93.44%, the approach was successful. The temporal and frequency information is captured by STFT, and the model’s capacity to learn hierarchical features is improved by its integration with 1D-CNN. Deep learning models’ interpretability is still an issue, though, especially in medical applications where getting the support of medical specialists requires being able to comprehend the reasons behind a choice. A Deep Fusion approach incorporating time, frequency, time-frequency, and entropy information was presented by Wang et al. (2021). High sensitivity (97.78%), specificity (97.42%), and accuracy (97.60%) were obtained with this method. The model is able to utilize complementary information from several areas because of the deep fusion of distinct properties. Deep learning models, however, frequently depend on vast quantities of labeled data, which can be a constraint when it comes to medical datasets because obtaining labeled samples can be resource-intensive.

In their seizure detection method, Zhao et al. (2023) utilized STFT and a Fully Connected Neural Network (FCNN) incorporating entropy and frequency domain characteristics. The reported accuracy was 88.14%, yet comprehensive information on sensitivity and specificity values was not provided. This absence of specific metrics restricts a thorough evaluation of the overall performance of the method. Furthermore, without detailed insights into the features employed, a comprehensive assessment of the strengths and weaknesses of the proposed strategy becomes limited.

With a sensitivity of 97% (SIFT) and 94% (ORB), our proposed approach shows promising results with fewer false negatives. With 95% as the specificity values for SIFT and ORB features the proposed work provides good classification performance with fewer false positives. Seizures can now be detected with an additional dimension because of the incorporation of image-based features. The suggested method makes a significant Contribution To The Field because of its high sensitivity and specificity as well as the interpretability of image-based elements. When these techniques are taken as a whole, a number of general patterns and issues become apparent. The trend toward integrating deep learning techniques—as shown in the methodologies of Wang et al. (2021) and Xuyang et al. (2021)—highlights neural networks capacity to identify intricate patterns in EEG data. Nonetheless, these models interpretability and explainability continue to be important factors, especially in medical applications where choices have an impact on patient care.

The selection of feature extraction methods is also crucial. Conventional signal processing methods, such as frequency analysis in Xuyang et al. (2021), and HOS in Sharma et al. (2020), offer important insights into the spectral and temporal properties of EEG signals. However, the suggested method’s use of image-based properties marks a divergence from traditional methods and creates opportunities for cross-disciplinary research. Even with the progress demonstrated by these techniques, seizure detection remains a chronic difficulty. Finding publicly accessible benchmark datasets containing documented seizure occurrences is difficult, which makes studies harder to replicate and compare. The creation of benchmark datasets and the standardization of assessment criteria can enable more thorough technique comparisons, promoting cooperation and quickening the field’s advancement.

Furthermore, there is a concern about how well these techniques are suited to manage variability among subjects and sessions. Since seizure patterns can differ greatly amongst individuals, it is essential to modify models to accommodate a wide range of patient demographics when using seizure detection systems in clinical settings. Subsequent investigations ought to concentrate on augmenting the applicability of models and tackling the obstacles presented by the intrinsic fluctuations in EEG signals. To sum up, this discussion’s comparative analysis of seizure detection techniques offers a thorough picture of the state of the art. Whether through conventional signal processing, deep learning, or novel feature extraction approaches, each method offers distinctive insights. Despite ongoing difficulties, the area’s aggregate advancements have positioned seizure detection as a dynamic, developing discipline that has the potential to have a big influence on clinical practice. The future of seizure detection will surely be shaped by ongoing cooperation, standardization, and the investigation of interdisciplinary approaches, ultimately helping those who have epilepsy and furthering the field of medical signal processing.

## Conclusion

In this work, our paper findings demonstrate the significant improvements in classification accuracy achieved through the proposed EEG signal preprocessing approach. The incorporation of GASF robust image formation, image-based feature extraction using SIFT and ORB, and feature selection has resulted in a classification accuracy of 96% using SIFT features and 94% using ORB features. These results are comparable to the state-of-the-art approaches in accuracy, precision, recall, F1-score, specificity, and AUC metrics. The implications of these findings are substantial, as they underscore the potential of the proposed methodology to advance the field of epilepsy detection and EEG-based diagnosis. The enhanced classification accuracy achieved through the proposed approach signifies a significant step forward in improving the diagnostic precision of EEG-based neurological disorders. The validation of the proposed system for EEG signals corrupted by Gaussian noise at different Signal to Noise Ratio (SNR) further highlights the robustness of the proposed approach. However, it is imperative to note that further research is essential to explore alternative feature extraction techniques, integrate deep learning models, investigate preprocessing parameters, and assess the generalizability of the proposed approach to larger and more diverse datasets. These avenues of future research hold promise for advancing the field of epilepsy detection and EEG-based diagnosis, ultimately benefiting individuals affected by epilepsy and contributing to the progression of medical signal processing.

## Data availability statement

Publicly available datasets were analyzed in this study. This data can be found here: https://www.upf.edu/web/ntsa/downloads/-/asset_publisher/xvT6E4pczrBw/content/2012-nonrandomness-nonlinear-dependence-and-nonstationarity-of-electroencephalographic-recordings-from-epilepsy-patients.

## Author contributions

PK: Conceptualization, Formal analysis, Investigation, Writing – original draft. SE: Validation, Visualization, Writing – review and editing. BB: Data curation, Formal analysis, Methodology, Writing – review and editing.
